# Accessing Care Across Cultures: Qualitative Insights Into the Reality of Informal Caregivers From Ethnically Minoritised Groups

**DOI:** 10.1111/hex.70426

**Published:** 2025-09-10

**Authors:** Anna Robinson‐Barella, Trinette Wayoe, Rosabella Louise Deakin, Charlotte Lucy Richardson

**Affiliations:** ^1^ School of Pharmacy Newcastle University Newcastle Upon Tyne UK; ^2^ Population Health Sciences Institute Newcastle University Newcastle Upon Tyne UK; ^3^ Newcastle Patient Safety Research Collaborative Newcastle Upon Tyne UK; ^4^ NIHR Applied Research Collaboration North East and North Cumbria Newcastle Upon Tyne UK

**Keywords:** caregiver, ethnic minority, ethnicity, health inequalities, qualitative

## Abstract

**Introduction:**

There remains limited research exploring the experiences of informal carers from ethnically minoritised groups, particularly to illustrate perceptions of caring roles and challenges they may face to address unmet needs. While barriers such as language, cultural expectations and discrimination are acknowledged in wider literature, little is known about how these influence caregiving experiences or access to services in practice. This work seeks to better describe the barriers and facilitators impacting carers from ethnically minoritised groups, as well as illustrate possible influences of culture and carer identity affecting this under‐researched population.

**Methods:**

Throughout June–July 2024, semi‐structured interviews were conducted with informal caregivers from ethnically minoritised groups (including: Pakistani, Black African, Indian, Arab, Chinese and Yemeni communities). Interviews were audio‐recorded and transcribed verbatim. Reflexive thematic analysis was used to develop themes. Ethical approval was obtained from Newcastle University Research Ethics Committee, ref: 2275/46476).

**Results:**

Fourteen carers of a variety of ages and ethnicities participated. Three overarching themes were developed from the data that centred on the following:

- Defining the role of a carer: people's journeys to becoming carers and links to roles as interpreters.
- Advocating for inclusive care: to receive accessible and understandable information.
- The impact of a person's wider community and culture upon their caring responsibility: culture and health are interlinked, and this influences the support that carers may need.

**Conclusion:**

Carers from ethnically minoritised groups have faced challenges with linguistic barriers and the paucity of healthcare services that are sensitive to cultural differences. Much of the work to date has focused on ‘formal’ caregivers and paid roles, as well as the experiences of people from ethnic *majority* groups. This study contributes unique perspectives to the current knowledge base and raises novel, learning points specific to supporting carers from ethnically minoritised groups. Future research could extend the conclusions from this work to specifically explore possible ethnic and cultural nuances in caring experiences.

**Patient or Public Contribution:**

An informal carer (David Black) was involved in the design of the study and its materials, and carers from various ethnic minority groups were involved. The NIHR INVOLVE guidelines and recommendations for carer involvement in research were followed.

**Trial registration:** Not applicable

## 1 Introduction

Current estimates in the United Kingdom have reported that one in five people undertake caring responsibilities [1]; however, given that many people do not recognise what they do as unpaid care, the actual number of carers could be significantly higher [2]. It is estimated that by 2037, the number of carers in the United Kingdom could increase to as many as 9 million [3]. It can be difficult to define the role of a carer [4]; within this work, a carer is defined as ‘anyone who looks after a family member, partner or friend who needs help because of their illness, frailty, disability, a mental health problem or an addiction and cannot cope without their support – the care they give is unpaid’ [5]. These carers are typically people who provide care to family members, with the most common arrangement being adult children providing support to parents across different households, or spouses caring for each other [6].

Evidence exists to support the association between higher numbers of people providing care amongst populations with greater levels of deprivation [7], those who are living with a disability [8] and people from ethnic minority groups [9]. This is more notable when these demographics intersect and a compounding of experiences is reported [10]. Amongst these groups, there remains a particular paucity of evidence around carer experiences from ethnic minority groups.

Existing literature has acknowledged the multifaceted challenges faced by people with a caregiving responsibility from ethnic minority groups; including associations with a greater number of health inequalities [11], reported language barriers or interpretation needs [11] and cultural expectations to provide care to family members [12]. There has also been a reported reluctance to receive care from nonfamily members, given that caring responsibilities were not viewed as separate from typical familial relationships [13]. Further, the term ‘carer’ itself cannot be directly translated into some languages, including (but not limited to) Bengali, Gujarati, Urdu and Punjabi [12, 14].

Ethnic and economic inequalities have exacerbated challenges in accessing health and care services, with one example being carer experiences of accessing COVID‐19 vaccines [9, 15, 16, 17, 18]. Findings from a Carers UK report, focused specifically on the challenges of carers from ethnic minority groups, stated an unmet need regarding the availability of healthcare professionals and social services for this group [19]. Despite the aforementioned challenges in their caring role, carers from minority ethnic groups provide a greater number of hours of care compared to their counterparts from majority ethnic groups [14, 20]. We have used the term ‘ethnic minority group’ throughout this work to describe people identifying as from communities which are a non‐majority, in accordance with the National Institutes of Health (NIH) ‘Racial and Ethnic categories and definitions for NIH diversity programmes and other reporting purposes’ [21] and the UK Office of National Statistics ‘Ethnic group, National identity and religion’ guides [22]; in the United Kingdom, this is non‐White communities [14].

It is recommended that health and social care interventions should be designed to be effective for different groups of people and in different contexts [23]. Carers and people from ethnically minoritised groups are both populations of particular focus within this [24], but there is a gap in bringing together understanding of the experiences of ethnic minority carers in relation to their access to health and social care services. While barriers such as language, cultural expectations and discrimination are acknowledged, little is known about how these shape lived caregiving experiences or access to services in practice.

### 1.1 Aim and Objectives

The aim of the study was to explore the perspectives of carers from ethnically minoritised groups, to establish an understanding of their lived experiences of accessing care services. The work seeks to better describe the barriers and facilitators affecting these individuals, as well as illustrate possible influences of culture and carer identity for this under‐researched population.

## 2 Methods

### 2.1 Recruitment and Sampling

The Consolidated criteria for reporting qualitative research (COREQ) checklist [25] was used for the reporting of this work (see Supporting Information S1: Material 1). Considering the potential of digital strategies to facilitate qualitative research, a blended approach was used to facilitate participant recruitment and data collection. Professional networks in the North of England (including research networks and patient and public involvement groups, via AR‐B and CLR), community spaces and charitable organisations served as recruitment hubs. Additionally, the research team shared the opportunity to participate via their professional social media accounts. Snowball sampling was used to recruit further participants. All participants who expressed interest were provided with a participant information sheet and consent form. Once written consent was collected, participants were enrolled in the study.

To be eligible to participate, people needed to identify as either being from an ethnically minoritised group living in the United Kingdom, according to the UK Office of National Statistics [22], or acting as a carer for a friend, family or community member living in the United Kingdom. Participants who had previously fulfilled this role but were no longer undertaking it due to reasons such as bereavement were also deemed eligible. There was no prior relationship between the researchers conducting interviews (T.W. and R.L.D.) and the participants.

Participants were not required to communicate in the English language: multilingual interpreters were available throughout the study for participants, where needed. Study documentation including the social media advert, information sheet and consent form were translated into languages common to the local community where the research was hosted including Arabic, Mandarin and Polish [26].

### 2.2 Data Collection

Between June and July 2024, two female pharmacy students (T.W. and R.L.D.) who received training in qualitative interviewing run by Newcastle University, in addition to research training from their undergraduate Master's degree, conducted semi‐structured interviews. Depending on participant preference, interviews were performed either in‐person, via telephone or online video calling (Zoom). Multilingual interpreters were available if required; author A.R.‐B. has expertise in this area [27] and professional interpretation services (with previous experience in qualitative research) were used as a strategy to mitigate against interpreter bias, ensure translation fidelity and capture the cultural contexts and nuances shared by participants [28, 29, 30, 31]. The interview topic guide (see Supporting Information S2: Material 2) covered issues identified from previous research [32, 33, 34], including ethnic minority participants' experience of caring and, engaging with, and accessing, health and care services and the potential barriers and facilitators to this. The guide was piloted with a carer before use. Questions were amended when needed as the interview stages progressed. Notes were made during interviews to help build topics of conversation relevant to participants.

### 2.3 Data Analysis

All interviews were audio‐recorded and transcribed using an automated transcription tool. These were accuracy checked by T.W. and R.L.D. Where interpreters were used, the transcripts included the questions asked in English by the researcher and the answers provided by the participant, which were translated into English by the interpreter. Files were encrypted, and audio files were destroyed following transcription. Interview data were anonymised at the point of transcription, and transcripts were checked for accuracy by T.W. and R.L.D. Participants did not provide comments on the transcripts.

Following the approach of reflexive thematic analysis by Braun and Clarke, two researchers (T.W. and R.L.D.) conducted an analysis of interview transcripts [35]. A detailed, close reading of the transcripts separately and then together enabled data familiarisation. Initial descriptive codes were identified; codes were then sorted into coding patterns and then, in turn, analytic themes. These themes were reviewed, refined and named once coherent and distinctive, and consensus was reached with the wider research team (A.R.‐B. and C.L.R.). The principle of constant comparison guided an iterative process [36]. Interview field notes enhanced the reflective analytical process; Microsoft Excel facilitated data management.

Our team deemed that data sufficiency and information power were reached after 14 interviews, with no new themes presenting at this point; hence, study recruitment ceased [37]. To ensure confidentiality throughout the research, when using direct quotes, participants were assigned non‐identifiable pseudonyms (P1, P2, etc).

### 2.4 Ethical Approval

Ethical approval was obtained by Newcastle University Research Ethics Committee (ref: 2275/46476).

### 2.5 Considerations When Reporting Participant Demographics

Due to the complex, multifaceted nature of defining ethnicity, collecting data around a person's ethnic group has been acknowledged as challenging [38]. There is no common definition of ethnicity, and, hence, this is often defined by subjective experiences of an individual [39]. The research team sought to collect and report multiple factors of a person's identity (including religion and the primary language spoken with the care recipient) to demonstrate the many layers that may influence a person's self‐defined ethnicity [33]. The National Institutes of Health and the UK Office of National Statistics informed the reporting of ethnicity [40, 41, 42].

### 2.6 Researcher Positionality and Reflexivity Statement

When conducting research relating to ethnicity, it is important to acknowledge the positionality and reflexivity of the wider research team [43]. Authors R.L.D. (a female undergraduate pharmacy student), A.R.‐B. (a female specialist clinical pharmacist and researcher with expertise in qualitative research and medicines inequity for marginalised groups) and C.L.R. (a female pharmacist and researcher with expertise in qualitative research and carer needs and experiences of health services) recognised their privilege as nonethnic minority UK citizens, as well as their roles as allies in tackling inequity in health and care provision. Author T.W. (a female undergraduate pharmacy student, self‐reported as being from an ethnically minoritised group) helped to ensure cultural appropriateness and sensitivity throughout the research process.

## 3 Results

### 3.1 Participant Demographics

Fourteen participants took part in this study (see Table 1); the majority provided care for a relative (*n* = 12, 86%) and two participants provided care for members of their community (*n* = 2, 14%). A diverse range of ethnicities were represented in this sample, with participants self‐reporting their ethnicity as British Pakistani (*n* = 6), Indian (*n* = 2), Black/Afro‐Caribbean (*n* = 2), Arab (*n* = 1), Yemeni (*n* = 1), Mixed‐Asian (*n* = 1) and Chinese (*n* = 1). Further diversity was seen in participant ages and in relationships between the carer and the care recipient.

**Table 1:** Participant demographics.

Participant number	Age (years)	Gender (self‐reported)	Ethnicity (self‐reported, verbatim)	Religion (self‐reported)	Care recipient (verbatim)	Interview format	Length of interview (min:s)	First language of care recipient
1	20–29	Female	British Pakistani	Muslim	Mother	Zoom	50:55	Urdu
2	20–29	Male	Black/Afro‐Caribbean	Catholic	Father	Zoom	12:15	English
3	70–79	Female	Indian	Jain, nonpractising	Sister and elder	Zoom	58:20	Elder relative— Gujarati sister— English
4	50–59	Male	Mixed Asian	Hindu	Mother	Telephone	19:56	Hindi, Afrikaans
5	70–79	Male	Indian	Humanist	Relative (unspecified)	Zoom	23:24	Gujarati
6	40–49	Male	Black/Afro‐Caribbean	Christian	Grandmother	Zoom	18:40	English
7	30–39	Male	Indian	Catholic	Community member	Zoom	29:02	English
8	30–39	Male	Arab	Muslim	Both parents	Zoom	53:28	Arabic
9	50–59	Female	British Pakistani	Muslim	Mother	Zoom	36:25	Punjabi
10	30–39	Female	Arab (Yemeni)	Muslim	Mother	Zoom	39:08	Arabic
11	20–29	Female	Chinese	Atheist	Mother	In‐person	20:39	Chinese
12*	60–69	Male	British Pakistani	Muslim	Wife	In‐person	87:39*	Urdu
13*	60–69	Male	British Pakistani	Muslim	Community member	In‐person	87:39*	Urdu
14*	70–79	Male	British Pakistani	Muslim	Wife	In‐person	87:39*	Urdu

*Note:* Key: * = participants involved in a group interview; interviews marked in bold = participants required an interpreter.

Nine interviews were conducted via video software (Zoom, *n* = 9), one by telephone (*n* = 1) and the remainder were carried out in‐person (*n* = 4). Interviews were conducted in June–July 2024 and ranged from 12 to 87 min in length. There were no refusals to partake, participant dropouts or repeat interviews. An interpreter was used to aid discussion with three participants (P12–P14).

**Figure 1:**
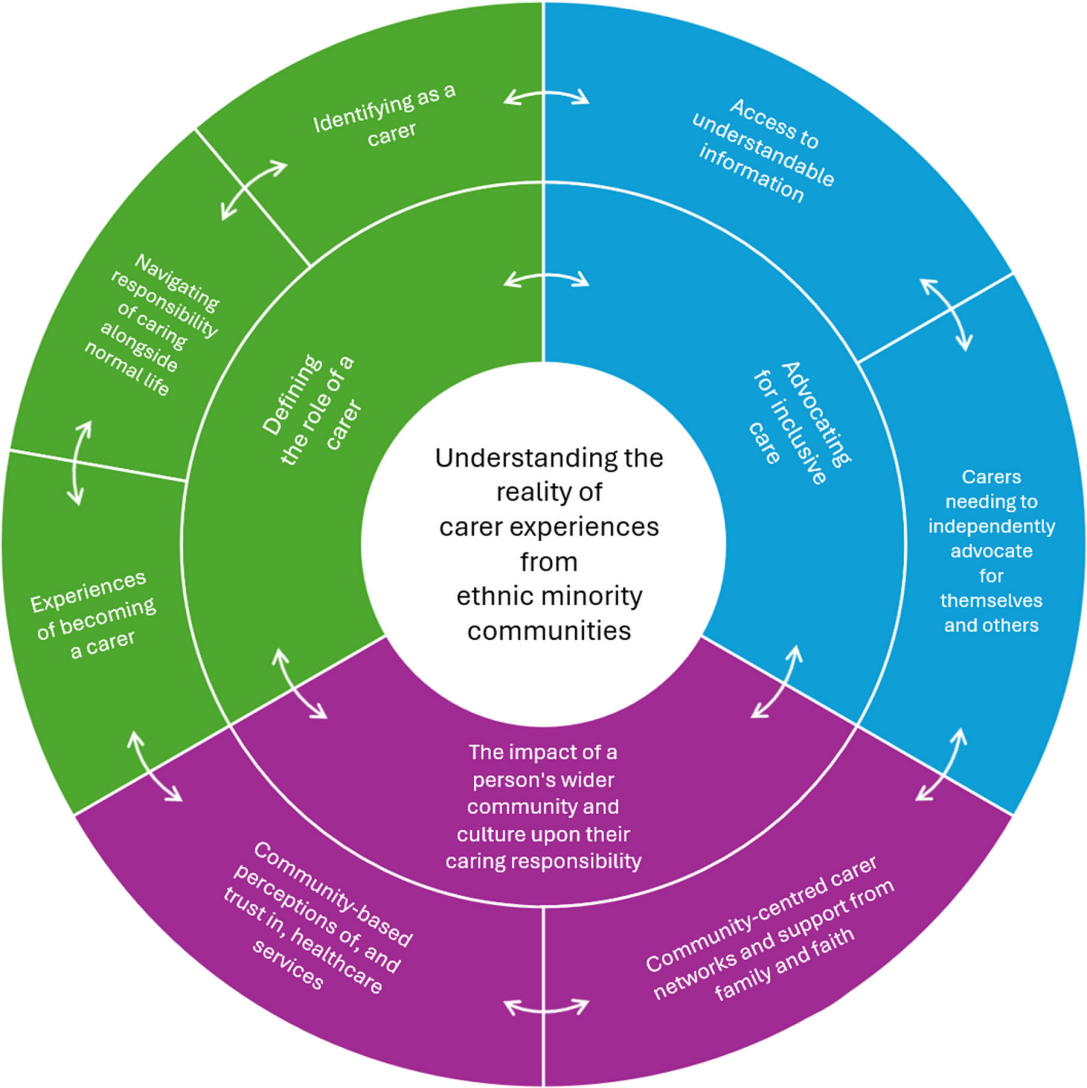
The themes and sub‐themes developed to summarise the lived experiences of being a carer from the perspectives of carers from ethnic minority groups. The arrows in the figure demonstrate the interconnectedness between the themes and sub‐themes to represent the wider picture of experiences shared by participants.

Many participants described experiencing complexities within their role as a carer, stating this was influenced by various interconnecting parts of their lives. These challenges and intersections informed the development of three themes which focused on the following: (1) defining the role of a carer, (2) advocating for inclusive care and appreciating (3) the impact of a person's wider community and culture upon their caring responsibility (Figure 1).

### 3.2 Theme 1: Defining the Role of a Carer

Experiences of becoming a carer: ‘So, I had to act as an interpreter as well as a family member’. (P11, a female carer of Chinese ethnicity, caring for her mother)

For some participants, a large part of assuming their role as a carer started from acting as a translator or interpreter for their parents or family members during medical appointments, ‘[my mum] doesn't speak [English] so we've always had to [interpret]’ (P9, a female carer of British Pakistani ethnicity, caring for her mother). From that point on, participants described an awareness of subtle shifts towards a role involving more caregiving; examples centred around a shift from reading and translating information during appointments to offering emotional and physical support for the individuals they care for.

Participants noted the unique challenges that come with acting as an interpreter for those they care for. Seemingly, healthcare professionals could also forget that carers attended appointments to be a source of emotional support, rather than practical support, with one participant mentioning that ‘because there is [an] official interpreter I [the carer] get kicked out of the room’ (P11, a female carer of Chinese ethnicity, caring for her mother). Other issues with communication stemmed from highlighting the differences between translation, interpretation and understanding. Participants who attempted to interpret for a family member or friend described additional challenges in the juggle to understand the medical jargon and terminology, whilst also expanding upon the finer details, during bilingual conversations. Coupled with interpreting without formalised training, this was regarded as an additional communication challenge. ‘There's no problem in speaking English. I can speak English, but sometimes I forget to, to talk or to explain things [when interpreting]’ (P14, a male carer of British Pakistani ethnicity caring for his wife). Equally, participants recognised that not all words had direct translations between languages or dialects, something which participants regarded as an essential criterion to build into the interpreter services offered. Consequently, participants reported these services to be inadequate due to differences in availability of languages and dialects offered.

Identifying as a carer: ‘Can I not just be a carer?’ (P1, a female carer of British Pakistani ethnicity caring for her mother)

Some participants acknowledged that the expectations of their wider communities and belief systems further impacted their identification of having a caring role. Many participants expressed that they took on their role as a carer without choice, but as something described as a cultural expectation within their community. ‘Honestly,… [caregiving] was just one of those things that you feel it's a duty, that has to be done’ (P10, a female carer of Arab [Yemeni] ethnicity caring for her mother). This viewpoint was echoed across participants, who described a strong sense of duty towards caring, suggesting that a caregiving responsibility was ingrained at a young age, possibly due to cultural expectations. Some carers described the neglect of communities to acknowledge the position of a carer, in part due to the presence of a cultural obligation to care. For example, ‘we've always done that role for our mom’ (P9, a female carer of British Pakistani ethnicity caring for her mother). This sense of obligation was presented an intrinsic part of participants' identity, which is shaped by cultural and/or religious narratives as well as personal experiences. For some participants, it appeared the expectation to take on a caring role was influenced by a cultural assumption that families should not seek external care provisions, ‘[in some communities] we wouldn't rely on external carers, they'd do it within the household’ (P10, a female carer of Arab [Yemeni] ethnicity caring for her mother).

Most participants felt that it was difficult to identify as a carer. One participant described a lack of a definition of the role within their community, compared to public perceptions of a formalised occupation, stating ‘[the carer role] is very misunderstood… because I myself would tell people I'm a carer, but I'm not you know, professionally recognised as a carer, or even formally recognised as a carer’ (P1, a female carer of British Pakistani ethnicity caring for her mother). Others echoed similar feelings, with some participants of older age citing that the terminology of being a carer may have not existed previously, which increased their struggle to identify with the role in the present day.

I have always been a carer, but we didn't know the term ‘carer’ back then. (P3, a female carer of Indian ethnicity caring for her sister and elder)

It was apparent that the relationship between a carer and their care recipient seemed to impact whether a person self‐identified as a carer, especially when providing care for relatives. Specifically, participants shared how they struggled to ‘draw a line and see the difference’ between acting as a family member or as a carer (P2, a male carer of Black/Afro Caribbean ethnicity caring for his father). This then impacted their understanding of who would be considered a carer, if not them, and at what point the difference between their intersecting ‘roles’ was identified. One male carer summarised this by stating ‘people don't know they're carers because they're supporting family. They think they are just supporting family, but they are indirectly carers’ (P5, a male carer of Indian ethnicity caring for his relative).

I'm more of a carer than a daughter when I'm doing it. (P9, a female carer of Indian ethnicity caring for her sister and an elder)

Navigating the responsibility of caring alongside their normal life: ‘Friday, he will have somebody else looking after his wife because he goes to the mosque’. (P12, an interpreter for a male carer of British Pakistani ethnicity caring for his wife)

For many carers, being able to participate in religious activities was regarded as an essential part of their ‘normal life’, especially as an opportunity to interact with their community and receive support. However, navigating the responsibility of finding alternative care arrangements for their relatives added to the existing stress and complexity of their caregiving.

Several participants shared similar views considering the duty and obligation when caring for someone. One participant shared that ‘all responsibility [of caring] is on you [as the carer]’ (P10, a female carer of Arab [Yemeni] ethnicity caring for her mother). Alike many other participants, this was attributed to feelings of anxiety and guilt when doing other things.

The most challenging aspect is trying to care for her, and also live my life. (P6, a male carer of Black African ethnicity caring for his grandmother)

Most participants shared the experience of needing to balance their caregiving with other elements of their life*. ‘*I have to project manage my time, otherwise it'll be chaos’ (P5, a male carer of Indian ethnicity caring for his relative). Similarly, some participants described turning down wider opportunities to ensure they were available to care. Participants suggested that this fed into their unspoken feelings of anxiety and stress when organising and arranging their daily activities.

Many participants described being the sole, primary carer and carrying the main responsibility for their relative. In turn, there were descriptions of this making them ‘less likely to reach out for support… because you know the person [being cared for] is dependent on you’ (P10, a female carer of Arab [Yemeni] ethnicity caring for her mother). Certain expectations and caregiving responsibilities were reported to be influenced by existing family structures, something that participants described as commonplace within their cultures, compared to others. One participant spoke of their efforts to find a balance in their carer‐life balance, by calling upon wider family members to share responsibilities and distribute specific roles amongst their siblings. They described ‘I might leave, and then [the care recipient] might have an hour or so before my brother and his wife arrive. She [the care recipient] does not spend very long by herself’ (P9, a female carer of British Pakistani ethnicity caring for her mother). On the other side, some participants chose to shoulder the caring responsibilities alone, which they described as a mechanism to protect their friends and relatives from the realities of caring worry; ‘Even though my brothers can [look after my parents], I would never allow them to do it… “what if my brother carried my dad and he fell or bumped him against something?”’ (P8, a male carer of Arab ethnicity caring for his parents).

I juggle multiple roles; I'm a student, I also work, I also have to care. (P1, a female carer of British Pakistani ethnicity caring for her mother)

### 3.3 Theme 2: Advocating for Inclusive Care

Access to understandable information: ‘It probably will be better if she can get all the information in the language she can understand’. (P11, a female carer of Chinese ethnicity caring for her mother)

To account for additional language requirements or variations in health literacy levels, participants shared the need to have information explained in understandable language and accessible formats. Several participants described how inclusive considerations around language and translated materials would provide benefit to their relatives, suggesting that health providers could ‘identify doctor surgeries in particularly mixed areas… and just have leaflets in a different language’ (P9, a female carer of British Pakistani ethnicity caring for her mother). However, it was also highlighted that there may be challenges in assuming written translated documentation to be the best ‘fix’.

My mom can't speak English. My mom hasn't had the benefit of an education so, actually, she's kind of illiterate in her own language as well, she can speak it fine but she can only read it a little bit… in terms of English, she can understand little bit, but not enough, and she's not very confident speaking. (P9, a female carer of British Pakistani ethnicity caring for her mother)

Most participants discussed the need to independently request information from healthcare providers, given a lack of information commonly shared about the benefits or support services available for undertaking a caregiving role. ‘Had I not been assertive and didn't know about [the paid benefit], how many more people might have asked for it and didn't get it?’ (P3, a female carer of Indian ethnicity for her sister and elder). This was echoed across a range of individuals, where participants had to go out of their way to seek answers and access to information regarding formalised support for those they care for. This was described as a difficult task when trying to organise already busy schedules. However, it could be compounded by experiencing microaggressions from individual healthcare professionals.

I don't know [if] it's…because I am Asian that the consultant refused to engage with me or include me in the consultations…? (P3, a female carer of Indian ethnicity for her sister and elder)

Carers needing to independently advocate for themselves and others: ‘I advocate on her behalf’. (P3, a female carer of Indian ethnicity caring for her sister and elder)

Participants described feeling like they were not being listened to, or being heard, when it concerned the care recipient's dietary requirements and/or religious preferences around treatment and medications. One participant stated that this caused distress due to their relative's requirements being overlooked and described how ‘you have to repeat lots, and you have to make sure they [healthcare professionals] understand the importance of this for them [person being cared for]’ (P5, a male carer of Indian ethnicity caring for his relative). In combination with the other stresses that accompany caregiving, this appeared to lead to an increased level of frustration towards healthcare providers. Despite this frustration, one participant described that their approach to advocacy centred on repetition.

My motto is to repeat everything, like lawyers do, so [the healthcare professionals] don't miss out. (P5, a male carer of Indian ethnicity caring for his relative)

Wider issues around discrimination and lack of cultural competency were discussed by participants when describing their experiences of advocating for care across primary and secondary healthcare services. One participant stated that ‘people are observing structural racism and discrimination within … not just the National Health Service, [but] wider society’ (P1, a female carer of British Pakistani ethnicity for her mother).

### 3.4 Theme 3: The Impact of a Person's Wider Community and Culture Upon Their Caring Responsibility

Community‐based perceptions of, and trust in, healthcare services: ‘We found out that within [minority groups], taboo is a big subject and that's caused a bit of a hindrance’. (P5, a male carer of Indian ethnicity for his relative)

Participants reflected that trust in the health system amongst the wider community could be influenced by acknowledging a person's culture, health and well‐being, and vice versa. Participants mentioned the role of traditional or herbal medicine, which plays a key part in health and wellness amongst some cultures, compared with prescribed treatments that were more typical of Western medicine. One carer mentioned ‘in the older generation, they have a lot of distrust [in Western treatment options]… they really want to go to that herbal or medical complimentary treatments’ (P1, a female carer of British Pakistani ethnicity for her mother). When traditional medicine was dismissed by healthcare professionals without acknowledging its cultural relevance for the individual person, participants cited feelings of disengagement and distrust from their care recipients being reluctant to follow treatment plans. Participants highlighted that, through culturally competent approaches, a better balance could be found.

[within minority groups] we have different ways of thinking in terms of ideas from all our countries and traditions, and they could easily help in the wider system. (P1, a female carer of British Pakistani ethnicity for her mother)

When interacting with a health professional, participants shared perceptions of greater trust with someone from a similar ethnicity or religious background to them and the person in receipt of care. One participant described how they ‘feel more comfortable talking to a Black doctor because they are like me’ (P6, a male carer of Black African ethnicity for his grandmother). Another participant recognised cultural‐specific nuances in communication styles, such as ‘it's not that they're nodding they're saying yes to that question. They're nodding the head that they are hearing you’ (P5, a male carer of Indian ethnicity for his relative). They described how misunderstandings in such instances led to frustration and less confidence in health services.

Community‐centred carer networks and support from family and faith: ‘I call on other family members and neighbours to help.’ (P6, a male carer of Black African ethnicity for his grandmother)

Many participants described the significance of community, family and faith within their support network to cope with caring responsibility. For many, this was viewed in a positive manner, with people expressing how they found community‐ and faith‐based resilience and comfort through activities such as prayer or interactions with others at places of worship. One participant shared how ‘I pray, and I find that gives me relief’ (P9, a female carer of British Pakistani ethnicity caring for her mother). However, there were some perspectives shared that demonstrated how these networks could still carry a stigma around discussing illness and caregiving. For example, one participant described a reluctance to seek support from within their community, citing that ‘I think it's a cultural thing… in a way, you don't acknowledge that you're that you're weak, or that you're going through something’ (P8, a male carer of Arab ethnicity caring for his parents).

Participants also described how the community expectation to care for elders, for some, came with feelings of guilt, a need to protect other family members around them, and a desire to not seek support, thus further perpetuating feelings of isolation. While this could be true of all carers, other cultural obligations appeared to exacerbate this triad. One carer stated how ‘we don't tell our parents or older relatives about any bad things that happen’ (P11, a female carer of Chinese ethnicity caring for her mother). This draws attention to the need for support networks that are sensitive to cultural differences. One participant cited the importance of carer support networks but recognised how cultural and/or religious tailoring is required for these to be most effective and inclusive.

The support networks should be there for all of us carers but, at the same time, some particular cultural needs are there too, which not everyone will relate to from all [religious] beliefs or [ethnic] descents. [Support] should be fulfilled with the particular community groups in mind, and that care should be provided. (P12, a male carer of British Pakistani ethnicity caring for his wife)

## 4 Discussion

This study explored the experiences and challenges of carers from ethnically minoritised groups. Specifically, the results considered aspects relating to a person's self‐definition and self‐identification as a carer, the importance of advocacy for inclusive and diverse care, and reiterated the significance of acknowledging intersections between caring, community and culture.

There was a recurring theme around caregivers struggling to identify with the terminology of being a carer; in part, this was possibly due to a person's cultural and/or familial expectations around providing informal care and whether this was understood to be a distinct role in keeping with the typical label of ‘carer’. Equally, services did not appear to be always be designed ‘with carers in mind’, where participants shared examples of barriers including: limited provision of interpreter services, challenges around the language used within consultations and services not being context‐specific to the diversity of the service users and their carers. Some carers described feeling a negative impact on their own health and well‐being due to their caring responsibilities, at times connecting this to cultural influences and a perceived expectation to not seek support outside of family or community units when it came to caring.

This study builds on the limited evidence on perspectives of carers from ethnically minoritised groups—a group that has been previously under‐represented and under‐reported in previous literature. Literature shows that the path to becoming a carer can be sudden and not predicted for most people [44, 45], but the findings from this work show that the journey appears to start earlier for those from ethnic minority groups compared to others, due to already having an active role in supporting health appointments by acting as an interpreter. This is echoed by research that found that children have had to be natural interpreters in their daily lives, which then can translate into a healthcare setting [46] and also appeared to be compounded in collectivist cultures [47].

People from ethnically minoritised groups have been reported to be at higher risk of microaggressions and discrimination when accessing health services, compared to people from ethnic majority groups [48, 49]. Microaggressions and examples of discrimination, related to ethnicity and race, that were raised by our participants echoed similar issues reported in the wider literature around carer experiences [50, 51]. However, much of the work to date has focused on ‘formal’ caregivers and paid roles; the bringing together of these two bodies of evidence in the context of informal carers is a novel finding from this study [52, 53]. A recent review, based in the United States, reported associations between unconscious biases and microaggressions, which lead to health inequalities [54], and so it could be suggested that carers from ethnic minority groups could themselves be at risk of health inequalities due to the burden of their caring role coupled with the presence of microaggressions.

Access to support across wider local communities was suggested as beneficial, as a way of offering support for individual ethnic and/or religious groups within an area, particularly where carers still tried to engage with cultural and/or religious obligations to support their own well‐being. This has previously been reflected in wider faith‐, community‐ and charity‐based suggestions to improve support networks within minoritised groups, specifically noting how places of worship could provide a place to offer support services in a familiar environment [55]. This is key, as previous studies have noted a link between support networks and improved outcomes influential in caring roles, such as health literacy [56].

While wider literature has suggested that informal caregiving roles are undertaken by females in the majority of cases, this study was unique in having a majority male sample and, in turn, unique in that the findings offer differing gender and cultural nuances versus the existing evidence base. It is also worth noting that a variation on culturally accepted gender roles could be impacted, as acknowledged in wider research, depending on generational factors—for example, second‐generation immigrants previously cited greater flexibility to these roles, compared to their first‐generation counterparts [57]. These factors could also affect suggestions on what support systems could be offered for future services, to facilitate better support [58].

An important consideration raised by participants in this study, echoing wider equity‐based health literature [59, 60], concerned language barriers and the challenges of establishing effective communication between healthcare professionals and people from ethnically minoritised groups. A dearth of evidence from wider health and care research has reported barriers to verbal communication experienced by people from ethnic minority groups, with associations between limited comprehension of language and delays in accessing healthcare services [61, 62], ineffective communication about medicines leading to poorer health outcomes and non‐adherence to treatments [15], and poorer patient, carer and professional satisfaction [63]. The use of interpreter services has been recognised to support information sharing and aid in the provision of culturally competent services for people from culturally and linguistically diverse groups [59, 64, 65]. However, participants in this study highlighted the specific importance of interpreters being inclusive of different dialects within a language, which extends beyond a ‘one size fits all’ approach to interpretation that has been previously inferred in the literature. The provision of appropriate, and culturally sensitive, written and translated information may also further facilitate design approaches for cultural and linguistic inclusivity, but such adoption should be made with caution given the recognised difficulties pertaining to formalised language [66].

## 5 Strengths and Limitations

This study was centred on the diverse voices of carers from ethnically minoritised groups and captured a range of cultural norms and caregiving experiences. There was diversity within the participants involved, including a variety of caregiving roles held by the participants, their ages and self‐reported ethnicities. The research team recognised that there were some limitations to this study. When reporting ethnicity, language is ever‐changing. At the time of writing this work, efforts were made by the research team to use the term ‘ethnically minoritised’ when describing a person's ethnic group; this was reported as a preferred term to use within the United Kingdom [67]; however, the authors acknowledge that this may be replaced with another preferred, and inclusive, term in the future [14]. While efforts were taken to ensure geographical representation across the United Kingdom, we recognise that these perspectives may not be generalisable for people from all ethnically minoritised groups residing elsewhere in the world—in particular, in relating to the navigation of different healthcare systems globally, and the intersectionality that may be individual to a particular ethnic group, culture or place. Future research may seek to explore international carer lived experiences to further determine these influences, as well as comparisons in experience between carers from ethnic minority and ethnic majority groups. By working with members of individual communities, using a community‐level approach, future research could further extend the conclusions from this work to more specifically explore possible ethnic and cultural nuances in caring experiences.

## 6 Conclusion

The experiences and challenges faced by carers from ethnic minority groups are influenced by cultural norms, and this can put pressure on individual carers to seek less support and/or not identify themselves as a carer. These carers may also face challenges around linguistic barriers and the absence of health care services that are sensitive to cultural differences and are welcoming of the caregiving role. Nevertheless, these individuals show perseverance and resilience throughout these challenges. This study provides insight to help develop culturally sensitive engagement with carers and start to direct the need for health and care services in the United Kingdom to be accessible to all carers.

## Author Contributions

**Anna Robinson‐Barella:** conceptualised the study and acted as a principal investigator and supervisor for Rosabella LouiseDeakin and Trinette Wayoe, and co‐lead on funding acquisition, methodology and project administration, data analysis and interpretation, and the writing of this manuscript with Charlotte Lucy Richardson. **Trinette Wayoe:** data curation, data analysis and interpretation, and the writing of this manuscript. He also ensured cultural appropriateness and sensitivity throughout the study. **Rosabella Louise Deakin:** data curation, data analysis and interpretation, and the writing of this manuscript. **Charlotte Lucy Richardson:** conceptualised the study and acted as a principal investigator and supervisor for Rosabella LouiseDeakin and Trinette Wayoe, and co‐lead on funding acquisition, methodology and project administration, data analysis and interpretation, and the writing of this manuscript with Anna Robinson‐Barella.

## Ethics Statement

Ethical approval was obtained by Newcastle University Research Ethics Committee (14/06/2024) (2275 46476).

## Consent

All participants provided informed consent to participate and were free to leave the study without consequence, ask questions or pause at any time.

## Conflicts of Interest

The authors declare no conflicts of interest.

## Supporting information

Supporting Information.

Supporting Information.

## Data Availability

The data that support the findings of this study are available upon reasonable request to the authors.
