# Supplementary material for: Accessing Care Across Cultures: Qualitative Insights Into the Reality of Informal Caregivers From Ethnically Minoritised Groups
Source: Health Expect. 2025 Sep 10;28(5):e70426. doi: 10.1111/hex.70426 (PMC12422111; doi:10.1111/hex.70426)
Supplement: Supplementary file 1 — Supporting Information. [file HEX-28-e70426-s002.docx]

**Item 1: COnsolidated criteria for REporting Qualitative studies (COREQ): 32-item checklist.**

| **Number** | **Item** | **Guide questions / description** | **Reported on manuscript page** |
| --- | --- | --- | --- |
| **Domain 1: research team and reflexivity** | | | |
| **Personal characteristics** | | | |
| 1 | Interviewer | Which author(s) conducted the interviews? | 7 |
| 2 | Credentials | What were the researcher’s credentials? *E.g., PhD, MD* | 7,9 |
| 3 | Occupation | What was their occupation at the time of the study? | 7,9 |
| 4 | Gender | Was the researcher male or female? | 7,9 |
| 5 | Experience and training | What experience or training did the researcher have? | 9 |
| **Relationship with participants** | | | |
| 6 | Relationship established | Was a relationship established prior to study commencement? | 7 |
| 7 | Participant knowledge of interviewer | What did the participants know about the researcher?  *E.g., reason for doing the research* | 7 |
| 8 | Interviewer characteristics | What characteristics were reported about the interviewer?  *E.g., bias, assumptions, reasons and interests in the research topic* | 7,9 |
| **Domain 2: study design** | | | |
| **Theoretical framework** | | | |
| 9 | Methodological orientation and theory | What methodological orientation was stated to underpin the study?  *E.g., grounded theory, ethnography, discourse analysis* | 6-8 |
| **Participant selection** | | | |
| 10 | Sampling | How were participants selected? *E.g., purposive, convenience, consecutive* | 6,7 |
| 11 | Method of approach | How were participants approached? *E.g., face-to-face, telephone, email* | 6,7 |
| 12 | Sample size | How many participants were in the study? | 8 |
| 13 | Non-participation | How many people refused to participate or dropped out (with reasons)? | Data not available |
| **Setting** | | | |
| 14 | Setting of data collection | How was the data collected? *E.g., home, clinic, workplace* | 7 |
| 15 | Presence of non-participants | Was anyone else present besides the participant and researcher? | 7,10 |
| 16 | Description of sample | What are the important characteristics of the sample? *E.g., demographic data* | 10 |
| **Data collection** | | | |
| 17 | Interview guide | Were questions and prompts provided by the authors? | 7 |
| 18 | Repeat interviews | Were repeat interviews carried out? If yes, how many? | 8 |
| 19 | Audio/visual recording | Did the researcher use audio or visual recording to collect the data? | 7,8 |
| 20 | Field notes | Were field notes made during/after the interview? | 7 |
| 21 | Duration | What was the duration of the interviews? | 10 |
| 22 | Data saturation | Was data saturation discussed? | 8 |
| 23 | Transcripts returned | Were transcripts returned to participants for comment/correction? | 7 |
| **Domain 3: analysis and findings** | | | |
| **Data analysis** | | | |
| 24 | Number of data coders | How many data coders coded the data? | 8 |
| 25 | Description of the coding tree | Did authors provide a description of the coding tree? | n/a |
| 26 | Derivation of themes | Were themes identified in advance or derived from the data? | 11,12 |
| 27 | Software | What software, if applicable, was used to manage the data? | 8 |
| 28 | Participant checking | Did participants provide feedback on the findings? | 7 |
| **Reporting** | | | |
| 29 | Quotations presented | Were participant quotations presented to illustrate the themes / findings? Was each quotation identified? E*.g., participant number* | 12-17 |
| 30 | Data and findings consistent | Was there consistency between the data presented and the findings? | 12-17 |
| 31 | Clarity of major themes | Were major themes clearly presented in the findings? | 12,14,16 |
| 32 | Clarity of minor themes | Is there a description of diverse cases or discussion of minor themes? | 12-17 |

**Item 2: Interview guide**

**Introduction**

Welcome to this research interview, my name is [X] and I will be talking to you today. Can I confirm your name please?

It is estimated that this interview will take up to 60 minutes (more likely 30-40 minutes). When the interview begins, it will follow a conversational style. There are no right and wrong answers to the questions we just want to know your thoughts and opinions. If you want to stop or pause the interview at any time, please just let me know. The interview will be audio-recorded so that we can accurately transcribe what you have told us. Once the interview recording has been transcribed and anonymised, the recording will be deleted. Anonymisation of the transcript will ensure that the participant cannot be identified.

We have shared with you are participant information sheet which details what the study entails and our consent form, do you have any questions regarding this? [answer any queries and then the participant signs the consent form].

**Starts recording and interview**

The semi-structured interview questions will be based around the following areas. The questions and lines of conversation will be guided by participant answers; further questions may be asked to explore perceptions/opinions in greater detail.

**Topics:**

1. **(Broader) Experiences being a carer (-- define carer, many may not view their ‘role’ as a ‘carer’, e.g. helping to care / support – and keep using the terminology they use)**. Examples: What does your role caring for them look like? What do you do to help them? Have you had any support from your friends and family? What about in the community/ place of worship?
2. **(Broad) Any personal experiences of accessing and receiving support from healthcare services broadly: in general / GP / pharmacy / other service.** Example: Can you tell us about your experiences visiting the GP/ Pharmacy? Hospital for yourself?
3. **(Narrowing) Experiences of accessing and receiving support from healthcare services in the context of being a carer (or whatever term they use in relation to Q1): in general / GP / pharmacy / other service.** Example: Can you describe any experiences you have had visiting the GP/hospital/pharmacy on behalf of or with the person you care for?
4. **(Narrow) Participant understanding of role of a carer - does ethnicity play a role?** Examples: How would you define the term carer? Do you feel your ethnicity has had any impact on this?
5. **(Focused) Access to care services and support networks (?clarify / prompt with this, e.g. going to pharmacy/GP/appointments/charities/community centres/places of faith)** Examples: Have healthcare providers been understanding of your cultural background? Why did you think that was? How did it make you feel?
6. **(Focused) Barriers to accessing care services – does ethnicity play a role in these?** Examples: Has anything specific made using healthcare services difficult? Have there been any challenges?
7. **(Focused) Facilitators to improve access to care services – does ethnicity play a role in these?** Example: Has there been anything that has improved your experiences?
8. **(Focused) ‘Blue sky’ thinking of the ‘ideal’, e.g. how services could be made more accessible / delivered in a way that better supports carers of people from ethnic minority communities.** Examples: In an ideal world, what do you think could be changed to improve your experiences? Do you feel there are any resources lacking that could be beneficial/ helpful?
